# Modulation of *Arabidopsis* and monocot root architecture by CLAVATA3/EMBRYO SURROUNDING REGION 26 peptide

**DOI:** 10.1093/jxb/erv360

**Published:** 2015-07-17

**Authors:** Nathan Czyzewicz, Chun-Lin Shi, Lam Dai Vu, Brigitte Van De Cotte, Charlie Hodgman, Melinka A. Butenko, Ive De Smet

**Affiliations:** ^1^Division of Plant and Crop Sciences, School of Biosciences, University of Nottingham, Leicestershire LE12 5RD, UK; ^2^Department of Biosciences, Section for Genetics and Evolutionary Biology, University of Oslo, N-0316 Oslo, Norway; ^3^Department of Plant Systems Biology, VIB, B-9052 Ghent, Belgium; ^4^Department of Plant Biotechnology and Genetics, Ghent University, B-9052 Ghent, Belgium; ^5^Department of Medical Protein Research, VIB, B-9000 Ghent, Belgium; ^6^Department of Biochemistry, Ghent University, B-9000 Ghent, Belgium; ^7^Centre for Plant Integrative Biology, School of Biosciences, University of Nottingham, Leicestershire LE12 5RD, UK

**Keywords:** *Arabidopsis thaliana*, auxin, *Brachypodium distachyon*, CLE26, root, wheat.

## Abstract

Plant roots are important for a wide range of processes, including nutrient and water uptake, anchoring and mechanical support, storage functions, and as the major interface with the soil environment. Several small signalling peptides and receptor kinases have been shown to affect primary root growth, but very little is known about their role in lateral root development. In this context, the CLE family, a group of small signalling peptides that has been shown to affect a wide range of developmental processes, were the focus of this study. Here, the expression pattern during lateral root initiation for several CLE family members is explored and to what extent CLE1, CLE4, CLE7, CLE26, and CLE27, which show specific expression patterns in the root, are involved in regulating root architecture in *Arabidopsis thaliana* is assessed. Using chemically synthesized peptide variants, it was found that CLE26 plays an important role in regulating *A. thaliana* root architecture and interacts with auxin signalling. In addition, through alanine scanning and *in silico* structural modelling, key residues in the CLE26 peptide sequence that affect its activity are pinpointed. Finally, some interesting similarities and differences regarding the role of CLE26 in regulating monocot root architecture are presented.

## Highlight

CLE26 plays an important role in regulating *A. thaliana* and monocot root architecture, and interacts with auxin signalling.

## Introduction

A small number of phytohormones have an impact on plant growth and development (Vanstraelen and Benkova, 2012;Davière and Achard, 2013; El-Showk *et al.*, 2013; Yoon and Kieber, 2013), but how this small number of chemical mediators can modulate the large number of physiological and biochemical responses required during growth and development remains an open question. Lately, however, it has become apparent that small signalling peptides provide an additional layer of control to steer growth and development (Murphy *et al.*, 2012; Czyzewicz *et al.*, 2013; Murphy and De Smet, 2014). Small signalling peptides generally range in size between four and 75 amino acid residues and most—but not all—are cleavage products from precursor peptides (Murphy *et al.*, 2012; Czyzewicz *et al.*, 2013). Several of these precursors are post-translationally modified prior to cleavage, and this post-translational modification can be critical for peptide activity and binding affinity for their receptor partners (Butenko *et al.*, 2009, 2014; Matsuzaki *et al.*, 2010; Matsubayashi, 2011; Murphy *et al.*, 2012; Shinohara and Matsubayashi, 2013).

The CLV3/EMBRYO SURROUNDING REGION (ESR)-related (CLE) peptide family is an ancient group of signalling peptides that has been shown to affect a wide range of developmental processes (Strabala *et al.*, 2006; Miyawaki *et al.*, 2013). The *Arabidopsis thaliana* CLE family is comprised of 32 peptides, each consisting of 12–13 amino acids, and containing a CLV3/ESR consensus sequence (Strabala *et al.*, 2006; Betsuyaku *et al.*, 2011). These CLE peptides are products of a larger precursor protein, which is translated and post-translationally modified prior to cleavage. However, only a few of the CLE peptides have been functionally characterized, and for most it is unknown which receptor(s) mediate(s) their signal (Strabala *et al.*, 2006; Shinohara and Matsubayashi, 2013). CLV3, the founding member of the CLE family, is perhaps the best characterized of the CLE peptides and exerts a crucial function as a mobile signal controlling the size of the *A. thaliana* shoot apical meristem (Clark *et al.*, 1995; Fletcher *et al.*, 1999; Ogawa *et al.*, 2008; Ohyama *et al.*, 2009; Shinohara and Matsubayashi, 2013). Since mature CLE peptides display a high degree of redundancy with known receptors (Strabala *et al.*, 2006; Wang and Fiers, 2010), it is likely that strict tissue-specific control of expression and/or the formation of co-receptor complexes provides signalling specificity. An example of this redundancy is seen for CLV3, CLE19, and CLE40, which all trigger consumption of the root apical meristem in *A. thaliana*, resulting in a short root phenotype, but only *CLE40* is actually expressed in the root apical meristem (Hobe *et al.*, 2003). The CLV3, CLE19, and CLE40 peptides all show an effect on the root meristem through a CLAVATA2 (CLV2)-dependent pathway (Fiers *et al.*, 2005), while CLV3 and CLE40 require CLV1 as part of a receptor complex to initiate the downstream signalling cascade (Ohyama *et al.*, 2009; Stahl *et al.*, 2013).

In land plants, the correct development of root architecture is vital for maximum uptake of water and minerals for growth. Root architecture differs greatly between species and can also be highly dynamic within a species if subjected to biotic or abiotic stress (Smith and De Smet, 2012). In addition to controlling primary root growth, developing lateral roots is another strategy which allows the plant to maximize the area over which nutrients are absorbed, and further allows the plant to anchor itself more firmly in the soil (Smith and De Smet, 2012). Lateral root primordia are formed from ~3 pairs of xylem pole pericycle cells from distinct cell files, which are primed by exposure to auxin in the basal meristem (Kurup *et al.*, 2005; De Smet *et al.*, 2007). Upon further exposure to auxin in the differentiation zone, the nuclei of these primed cells move toward a central point, and the cells divide asymmetrically to form two central small daughter cells, and two distal large daughter cells—the stage 1 primordium (De Smet *et al.*, 2007). These cells divide asymmetrically in a strictly regulated manner through seven further stages, during which the primordium pushes through the endodermis, epidermis, and cortex, and becomes the emerged lateral root (Péret *et al.*, 2009; Lavenus *et al.*, 2013).

Here, CLE peptides, which—based on their expression profile—are likely candidates for regulating root architecture, were identified. To explore further their *in vivo* involvement in root development, their root growth response to synthetic peptide treatment was assessed. Based on the results, one member of the CLE family, namely CLE26, subsequently became the focus of the study, and its function and relationship to auxin signalling and response in *A. thaliana* were explored. By employing CLE26 peptide treatment on roots of *Brachypodium distachyon* and wheat, to what level there is functional conservation between *A. thaliana* and monocots was also investigated.

## Materials and methods

### Plant lines

*A. thaliana* lines expressing the β-glucuronidase gene (*GUS*) under the control of *CLE* peptide promoters were obtained from the Nottingham Arabidopsis Stock Centre (NASC) and/or provided by Jennifer Fletcher (Jun *et al.*, 2010) (Supplementary Table S1 available at *JXB* online). The following previously published lines were used: *arf7 arf19* (Okushima *et al.*, 2007), *pDR5::GUS* (De Smet *et al.*, 2007), *35S:DII:VENUS* (Brunoud *et al.*, 2012; Vernoux *et al.*, 2011), and *pPIN1::PIN1*:GFP (Benková *et al.*, 2003). The *cle26-1* (N689781) SALK T-DNA line was genotyped by PCR using primers designed by the T-DNA Primer Design Tool (signal.salk.edu) (*cle26-1* Forward, ACCCATTTTGTGTTTTTGCAC; *cle26-1* Reverse, ATTATACGCGTGGACCACTTG; and SALK LBb, ATTTTGCCGATTTCGGAAC) and using the following PCR conditions: 94 °C 5min, 40× (94 °C 30 s, 60 °C 30 s, 72 °C 1min), 72 °C 5min.

### Growth conditions

*A. thaliana* and *B. distachyon* seeds were surface sterilized by immersion in 70% ethanol for 30 s, followed by immersion in 25% bleach for 20min, and were then vernalized at 4 °C. The *A. thaliana* seedlings were grown at 20–22 °C, with 24h daylight under fluorescent lamps [150 μM (m^2^)^–1^ min^–1^] on 12×12cm square Petri dishes containing 50ml of half-strength Murashige and Skoog (1/2 MS) agar [2.154g of MS medium (Duchefa), 0.1g of myo-inositol (Sigma-Aldrich), 0.5g of MES (Sigma-Aldrich) 1% (w/v) agar (Sigma) per litre of distilled water] containing the appropriate concentration of peptide diluted in water until 12 days after germination (DAG). Wheat seeds were surface sterilized by immersion in 5% hypochlorite for 15min, before washing three times in water. Wheat and *B. distachyon* seedlings were grown at 20–22 °C, with 24h daylight in 100ml boiling tubes containing 20ml of 1/2 MS agar with the appropriate concentration of peptide until 10–12 DAG. For GUS analyses, seedlings were grown at 20–22 °C, with 24h daylight on 8cm radius round Petri dishes containing 20ml of 1/2 MS agar until 6 DAG. For quantitative PCR (qPCR) analyses, *A. thaliana* seedlings were grown at constant light conditions on 1/2 MS agar for 7 DAG and then transferred to 1 μM CLE26p for 24h, or at 5 DAG to 1 μM 1-naphthaleneacetic acid (NAA). For green fluorescent protein (GFP) studies, seeds were surface sterilized by immersion in 70% ethanol for 30 s, followed by 20min immersion in 20% bleach (final concentration 1% hypochlorite). Sterilized seeds were washed three times in sterile distilled water, before vernalizing for 24h at 4 °C. Sterile, vernalized seeds were germinated and grown on 1/2 MS medium [0.01% myo-inositol (Sigma-Aldrich), 0.05% MES (Sigma-Aldrich), and 1% (w/v) agar] until 5 DAG.

### Synthetic peptides

Synthetic peptides were ordered from GenScript (www.genscript.com/) and were used at the following purities: CLE1/4p (98.8% or 98.2%), CLE7p (94.8% or 88.9%), CLE27p (71.2% or 73.6%), CLE26p (94,2, 89.6, 84.6, or 99.7%), mCLE26p 1A (92.1%), mCLE26p 2A (91.6%), mCLE26p 3A (99.4%), mCLE26p 4A (98.2%), mCLE26p 5A (98.6%), mCLE26p 6A (84.9%), mCLE26p 7A (94.3%), mCLE26p 8A (99.1%), mCLE26p 10A (97.5%), mCLE26p 11A (94.3%), and mCLE26p 12A (94.7%). All peptides used were diluted to a 10mM stock solution, correcting for purity [volume required for 10 mM×(purity/100)], and sequentially diluted to working concentrations prior to use. Stock and working solutions were stored at –20 °C.

### Root architecture measurements

Emerged *A. thaliana* or *B. distachyon* lateral roots were counted using a dissection microscope. *B. distachyon* was removed from agar tubes by heating in a 95 °C water bath until the agar was melted, and mounted in 12×12cm plates in sterile, distilled water prior to analysis. Photos of the plates were taken using a digital camera mounted on a fixed stand before and after counting lateral roots. Primary root length and lateral root numbers were measured by use of FIJI (Schindelin *et al.*, 2012) and analysed in Excel. Lateral root density was calculated as the total number of emerged lateral roots/total primary root length.

### GUS screening

Seedlings at 10–12 DAG were stained in GUS staining solution (0.05M sodium phosphate buffer, 5mM potassium ferrocyanide, 5mM potassium ferricyanide, 1mM 5-bromo-4-chloro-3-indolyl-β-d-glucuronide) until the stain was visible under a dissection microscope, before stopping the reaction by immersion in 70% ethanol for 5min. The seedlings were then cleared by incubation in acidified methanol [20% (v/v) methanol, 4% (v/v) HCl] for 15min, followed by incubation in alkalinized ethanol [60% (v/v) ethanol, 7% (w/v) NaOH]. The ethanol concentration was then gradually decreased by 15–20min incubations in each of 40, 20, and 10% ethanol, before storage and analysis of the expression pattern in distilled water (Malamy and Benfey, 1997). Stained seedlings were imaged using a Leica DMRB binocular microscope and Leica Application Suite (Leica). When necessary to improve figure quality, brightness and contrast of the images were modified using Photoshop and, in such cases, all related figures were treated the same.

### GFP analysis

Seedlings grown in the presence of CLE26p or 5-day-old seedlings transferred to fresh plates containing CLE26p were analysed by confocal microscopy (Leica TCS SP5) at 5 DAG or at different time points (0, 6, and 24h), respectively.

### RNA extraction, cDNA synthesis, and (q)RT–PCR analysis

Total RNA was isolated with Trizol reagent (Invitrogen) or by using the RNeasy Mini Plant Kit (Qiagen) according to the manufacturer’s instructions. Poly(dT) cDNA was prepared from 2mg of total RNA with Superscript II/III reverse transcriptase (Invitrogen) and quantified on an LightCycler 480 apparatus (Roche Diagnostics) with the SYBR Green I Master kit (Roche Diagnostics) according to the manufacturer’s instructions using gene-specific primers (CLE26 FW 5′-ACCATTCCCTTCGTCTCCA-3′ and CLE26 REV 5′-CGTCGTTCCTTGAACCATCT-3′). Three biological repeats were performed on a pool of seedlings and all individual reactions were done in triplicate. Graphs show one representative analysis. Data were analysed with qBase (Hellemans *et al.*, 2007) and normalized to *EEF1a4* (EEF1α4_FW 5′-CTGGAGGTTTTGAGGCTGGTAT-3′ and EEF1α4_REV 5′-CCAAGGGTGAAAGCAAGAAGA-3′) and/or *ARP7* (ARP7_FW 5′-ACTCTTCCTGATGGACAGGTG-3′ and ARP7_REV 5′-CTCAACGATTCCATGCTCCT-3′). To detect CLE26.1 and CLE26.2 splice variants, RNA was extracted with RNeasy (Qiagen), cDNA was prepared with an iScript cDNA Synthesis Kit (Bio-Rad), and specific primers (CLE26_F ATGCGAAATAACCATTCCCTTC and CLE26.2_AS TTATGCTCTTCCTGGTGGTCG or CLE26.1_AS TCATACAGAAACATCCAAGACACAT) were used for PCR [94 °C 3min, 40× (94 °C 30 s, 55 °C 30 s, 72 °C 1min), 72 °C 7 min].

### Genevestigator analyses

Genevestigator (www.genevestigator.com) was used to assess transcriptional regulation of *CLE* genes for which microarray probes are available, using settings with a fold change of 2 and a *P*-value of 0.05.

### Phylogenetic analyses

Full-length *A. thaliana* CLE peptide precursor sequences were obtained from Uniprot for all known *A. thaliana* CLE peptides. These sequences were used to BLAST for similar sequences in *Zea mays*, *Oryza sativa, B. distachyon*, *Brassica rapa*, *Selaginella moellendorffii*, and *Physcomitrella patens* using Phytozome v9.1 (Goodstein *et al.*, 2011). The indicated sequences were aligned with *A. thaliana* CLE peptide precursors to identify the conserved CLE region, and phylogenetic trees were built in CLC Workbench using the 12–13 amino acid conserved domains. The mature 12 amino acid sequences from *A. thaliana* and *B. distachyon* were further used to create a Weblogo alignment using WebLogo3 (Crooks *et al.*, 2004).

### Structure prediction

The precursor protein was submitted to PHYRE for standard tertiary structure prediction and analysis (www.sbg.bio.ic.ac.uk/phyre2) (Kelley and Sternberg, 2009). The predicted secondary structure, model ranking, and relevant extra figures are shown in the Supplementary data at *JXB* online. Rotamers of Arg5 and Asp8 (Dunbrack, 2002) were explored and structural figures produced using Chimera v.1.5.3 (Pettersen *et al.*, 2004).

## Results and discussion

### CLE peptide expression is regulated by hormones and environmental triggers

Root architecture is controlled by hormonal and environmental cues (Smith and De Smet, 2012; Tian *et al.*, 2014). For example, auxin positively regulates several aspects of lateral root initiation, primordium development, and emergence (De Smet, 2012; Lavenus *et al.*, 2013), and abscisic acid (ABA) is crucial for salt-regulated root growth dynamics and acts—for instance—on meristem activation (De Smet *et al.*, 2003; Ding and De Smet, 2013; Duan *et al.*, 2013). Also, in response to various nutrient deficiencies, the root system displays high plasticity (Gruber *et al.*, 2013; Tian *et al.*, 2014). It is therefore not unexpected that gene expression of factors regulating root architecture is under hormonal and environmental control, and a number of reports suggest this to be the case for *CLE* expression. Specifically nitrate and phosphate appear to affect *CLE* expression strongly in various species during the regulation of root architecture and/or nodulation, as do cytokinin and gibberellin (Okamoto *et al.*, 2009; Funayama-Noguchi *et al.*, 2011; Reid *et al.*, 2011; Mortier *et al.*, 2012; Araya *et al.*, 2014; Bidadi *et al.*, 2014; Lim *et al.*, 2014). To assess comprehensively the influence of hormones, nutrients, and abiotic stresses on the regulation of *CLE* expression in *A. thaliana*, available microarray data sets were probed. The combined analysis for those *CLE* genes—for which data were available and focusing on hormones and environmental triggers that had a significant effect—is shown in Table 1. Strikingly, *CLE2* and *CLE6* were mainly down-regulated by hormones, nutrients, and stress, while *CLE12* was mainly up-regulated. *CLE3*, *CLE26*, *CLE27*, *CLE41*, *CLE44*, and *CLE46* showed variable responses to the selected stimuli. For example, *CLE26*, *CLE41*, and *CLE44* are down-regulated by salicylic acid, while ABA and auxin up-regulated *CLE27*, *CLE41*, and/or *CLE44*. In conclusion, it indeed appears that environmental cues and hormone levels effect *CLE* expression, possibly during primary and lateral root growth and development. However, these global transcript data do not provide any information on where and when *CLE* genes relevant for root architecture are expressed.

**Table 1.** Hormonal and environmental control of CLE expression

	Hormones					
	Brassinosteroids	ABA	Auxin	Jasmonic acid	Salicyclic acid	
*CLE2*	−	−	−			
*CLE3*						
*CLE6*	−					
*CLE12*		+	−	+		
*CLE26*					−	
*CLE27*			+			
*CLE41*		+			−	
*CLE44*		+	+		−	
*CLE46*						
	Nutrients					
	Glucose	Iron deficiency	Nitrate	Phosphate deficiency	Sulphur deficiency	
*CLE2*	−	−	+		−	
*CLE3*				+		
*CLE6*	−	−		−		
*CLE12*	+			+	+	
*CLE26*						
*CLE27*						
*CLE41*	+					
*CLE44*		+				
*CLE46*						
	Stresses					
	Anoxia	Cold	Drought	Heat	Hypoxia	Salt
*CLE2*				−	−	
*CLE3*						
*CLE6*	−	+	−	−	−	
*CLE12*		+	+			+
*CLE26*						
*CLE27*						
*CLE41*				−		
*CLE44*		+	−			
*CLE46*			+			

+, significant up-regulation; –, significant down-regulation.

### Primary and lateral root *CLE* expression patterns in *A. thaliana*

The expression pattern for several *CLE* genes has been described previously (Sharma *et al.*, 2003; Geier *et al.*, 2008; Hirakawa *et al.*, 2008; Jun *et al.*, 2010; Depuydt *et al.*, 2013). However, to evaluate which CLE peptides might play a role in mediating *A. thaliana* root architecture comprehensively and comparably, the expression patterns of 20 *CLE* genes in the primary root tip and during lateral root development were profiled using previously published *pCLE::GUS* fusions (Jun *et al.*, 2010) under the growth conditions used here. This analysis revealed distinct expression patterns, both in the root tip and during early stages of lateral root development (Supplementary Figs S1–S4 at *JXB* online). The majority of the observations are in agreement with an earlier report (Jun *et al.*, 2010); however, expression of *pCLE3::GUS*, *pCLE16::GUS*, and *pCLE17::GUS* was not observed in the vasculature. Given that the expression of *CLE* genes can be subject to environmental control (Table 1), the differences in expression observed could potentially be due to variations in growth conditions.

**Fig. 1.**
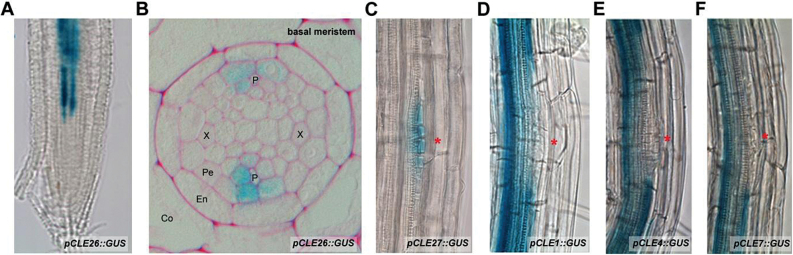
*CLE* expression visualized through *pCLE::GUS* lines. (A, B) *pCLE26::GUS* in the primary root tip: (A) whole mount; (B) transverse section in the basal meristem. (C–F) *pCLE::GUS* during early stages of *A. thaliana* lateral root development: (C) *pCLE27::GUS*; (D) *pCLE1::GUS*; (E) *pCLE4::GUS*; (F) *pCLE7::GUS*. A red asterisk indicates the position of the lateral root primordium. Seedling age, 5–7 d after germination.

Based on the observed *CLE* expression patterns, a subset of CLEs was used for further analyses. Expression of *pCLE6::GUS*, *pCLE22::GUS*, *pCLE25::GUS*, *pCLE26:: GUS*, and *pCLE27::GUS* was observed in the basal meristem, with *CLE6*, *CLE22*, *CLE25*, and *CLE26* expression seemingly restricted to the stele and with *CLE26* being the most strongly expressed (Fig. 1A; Supplementary Fig. S1 at *JXB* online). The latter expression patterns may indicate a role in lateral root patterning via establishment of the vascular pattern and/or priming of pericycle cells (De Smet *et al.*, 2007; Parizot *et al.*, 2008, 2012). During the early stages of lateral root development, *pCLE27::GUS* was specifically expressed in the asymmetrically dividing pericycle cells (Fig. 1C). Based on this expression pattern (in the core of the lateral root initiation site), it was hypothesized that CLE27 could be a positive regulator of early stages of lateral root development. In contrast, during lateral root initiation and development, *pCLE1::GUS*, *pCLE4::GUS*, and *pCLE7::GUS* were expressed in the vasculature and pericycle, but excluded from the developing primordium (Fig. 1D–F). Based on these expression patterns (excluded from the lateral root initiation site), it is hypothesized that these CLEs may be negative regulators of early stages of lateral root development.

### Synthetic CLE peptides affect *A. thaliana* primary and lateral root growth and development

**Fig. 2.**
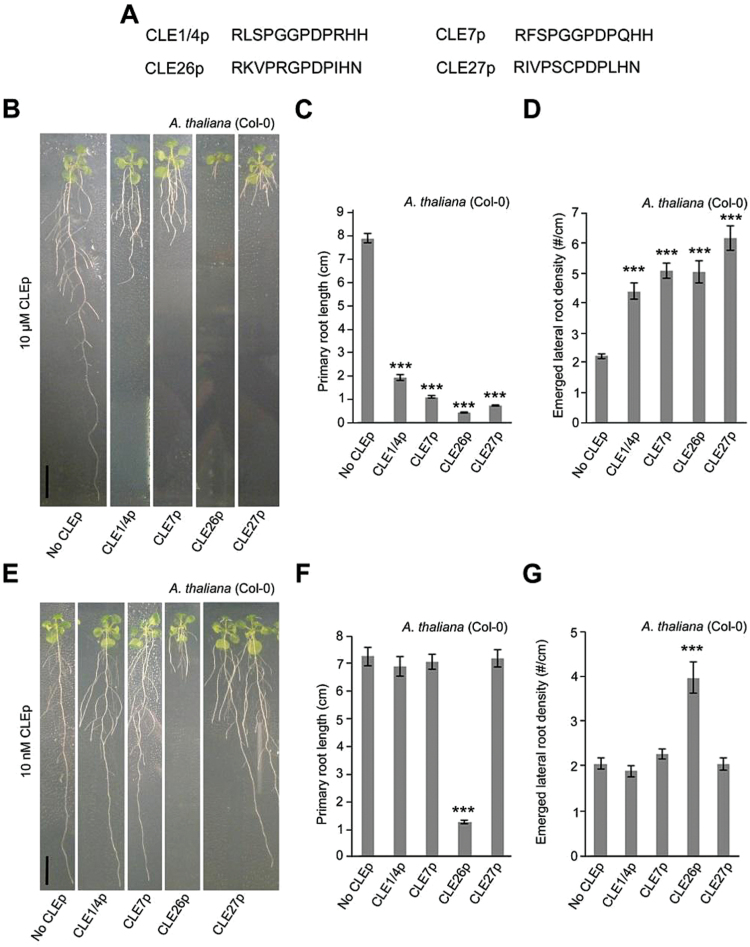
CLE peptide treatment of *A. thaliana*. (A) Sequence of synthetic CLE peptides used. (B–G) Treatment of wild-type seedlings with 10 μM (B, C) or 10nM CLE peptide (E–G). Representative pictures of CLE26p-treated wild-type seedlings at 12 d after germination (B and E). Quantification of primary root length (C and F) and emerged lateral root density (D and G) for CLE26p-treated wild-type seedlings. The bar graphs indicate the mean ±SE. Statistical significance (Student’s *t*-test) compared with the no peptide treatment is indicated: ***P<0.01. Scale bar=1cm. (This figure is available in colour at *JXB* online.)

To characterize further the involvement of *CLE* genes with an interesting lateral root-associated expression pattern and/or a regulation by hormonal or environmental triggers, chemically synthesized CLE peptides (CLEps) corresponding to the predicted products of the respective *A. thaliana CLE* genes (AtCLEp, referred to as CLEp) were used. CLE1p, CLE4p, CLE7p, CLE26p, and CLE27p were selected but, since CLE1 and CLE4 have the same mature peptide sequence, they were represented by one synthetic peptide (CLE1/CLE4p) (Fig. 2A). Based on their expression patterns, a repressive (CLE1, CLE4, and CLE7) or inductive effect on lateral root development (CLE26 and CLE27) was hypothesized for the selected peptides. To test the biological activity of the chemically synthesized peptides, initially a high concentration (compared with likely normal physiological conditions) of 10 μM CLEp was applied to wild-type *A. thaliana* seedlings. This revealed a significant effect of all assayed CLE peptides, namely decreased primary root length (between a 76% and 94% decrease), decreased lateral root number (between a 57% and 88% decrease), and increased lateral root density (between a 98% and 179% increase) (Fig. 2B–D; Supplementary Fig. S5 at *JXB* online). The decrease in primary root length was predictable, since overexpression of *CLE* genes or application of chemically synthesized CLE peptides often results in consumption of the root apical meristem and/or a short primary root, and is possibly a non-specific response to high concentrations of exogenously applied peptide (Casamitjana-Martinez *et al.*, 2003; Fiers *et al.*, 2005; Kinoshita *et al.*, 2007; Jun *et al.*, 2010; Depuydt *et al.*, 2013). In addition, the increased lateral root density by CLE1, CLE4, and CLE7 is in contrast to the hypothesized effect, namely a decrease in lateral root development. It is, however, possible that the increased lateral root density is a consequence of the dramatically reduced primary root growth.

**Fig. 3.**
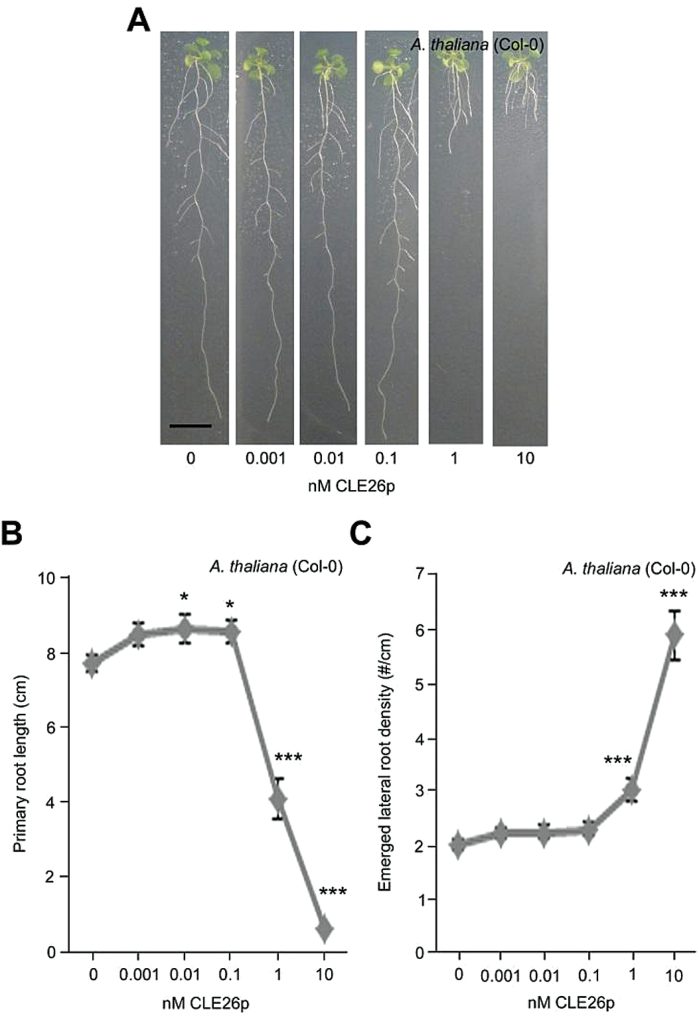
CLE26p concentration gradient on *A. thaliana*. (A) Representative pictures of CLE26p-treated wild-type seedlings at 12 d after germination. (B, C) Quantification of primary root length (C) and emerged lateral root density (D) for CLE26p-treated wild-type seedlings. The graphs indicate the mean ±SE. Statistical significance (Student’s *t*-test) compared with no peptide treatment is indicated: ***P<0.01; *P<0.05. Scale bar=1cm. (This figure is available in colour at *JXB* online.)

Subsequently, it was assessed whether these chemically synthesized CLE peptides also affected root architecture at a lower, more physiologically relevant concentration (10nM). Analyses of *A. thaliana* seedlings grown on 10nM CLEp revealed that only those seedlings grown on CLE26p displayed a significant 83% decrease in primary root length, a 72% decrease in lateral root number, and a 94% increase in lateral root density compared with the control (Fig. 2E–G; Supplementary Fig. S5 at *JXB* online). However, there was no obvious effect of CLE1p, CLE4p, and CLE7p on primary root length and neither did these seedlings display a reduced lateral root density. A dose–response analysis further indicated that CLE26p is able to restrict primary root growth and increase lateral root density in *A. thaliana* at a minimum concentration of 1nM (Fig. 3A–C). This is a similar activity threshold to other peptides, such as, for example, RALF, which is also active in the nanomolar range (Pearce *et al.*, 2001), and TDIF (CLE41/CLE44), which is active in the picomolar range (Sawa *et al.*, 2006). Surprisingly, the present CLE26p application data (at higher concentrations) are not in agreement with earlier observations based on overexpression of *CLE26* (Strabala *et al.*, 2006), but correspond to another report that showed that 19 CLE peptides are able to induce a short root phenotype (Kinoshita *et al.*, 2007). In agreement with the present results, the latter study also showed that among all CLE peptides tested, CLE26p is the most effective one in inducing the short-root phenotype in *A. thaliana*. Intriguingly, CLE26p resulted in a subtle, but significant, increase in primary root length at a concentration of 0.1nM and 0.01nM (Fig. 3B), and it is possible that the previously reported *CLE26* overexpression lines (which could be mild overexpressors) (Strabala *et al.*, 2006) capture this. Taken together, the results suggest that CLE26 plays a role in *A. thaliana* primary and lateral root growth and development.

### *CLE26* is expressed at the phloem pole

To gain insight into the effect of CLE26p in relation to its expression pattern at cellular resolution, transverse root sections of *pCLE26::GUS* seedlings were analysed. This revealed that *CLE26* is expressed in the stele at the phloem pole (Fig. 1B), which is in agreement with a genome-wide expression profiling of xylem and phloem–cambium isolated from the root hypocotyl of *A. thaliana* where a phloem–cambium bias was reported (Zhao *et al.*, 2005). It was recently proposed that CLE45, BAM3, and BRX might interact to guide the proper transition of protophloem cells from proliferation to differentiation, which could determine the growth capacity of the root meristem (Depuydt *et al.*, 2013; Rodriguez-Villalon *et al.*, 2014), and it is possible that CLE26 has a similar function. To support this, fewer cells expressing the protophloem marker *pAT2G18380::GFP* (S32; Lee *et al.*, 2006) were observed in CLE26p-treated root tips (Supplementary Fig. S6 at *JXB* online). It is, however, unlikely that CLE26 signals through the same pathway as CLE45 as a *bam3* mutant is equally sensitive to CLE26 as the wild type (Depuydt *et al.*, 2013), ruling out BAM3 as a putative CLE26 receptor. However, CLE26 displayed similar strong binding affinity for BAM1 and BAM2, and moderate affinity for CLV1 and CLV2 (Guo *et al.*, 2010), making these receptors good candidates for mediating the CLE26 phenotype.

### CLE26 peptide sequence–activity analyses identify key amino acids

**Fig. 4.**
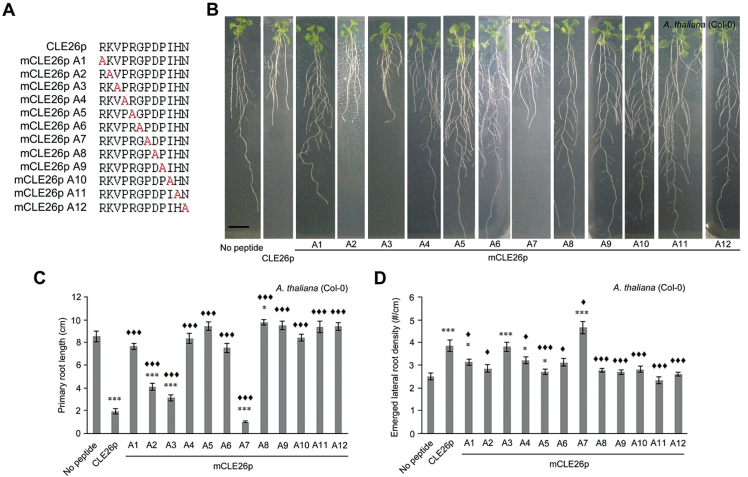
CLE26p alanine scanning on *A. thaliana*. (A) Sequence of synthetic CLE peptides used. (B) Representative pictures of mCLE26p-treated wild-type seedlings at 12 d after germination. (C, D) Quantification of primary root length (C) and emerged lateral root density (D) for mCLE26p-treated wild-type seedlings. The bar graphs indicate the mean ±SE. Statistical significance (Student’s *t*-test) compared with no peptide (*) and to CLEp treatment (♦) is indicated: ***/♦♦♦*P*<0.001, */♦*P*<0.05. Scale bar=1cm. (This figure is available in colour at *JXB* online.)

Given that application of low—possibly physiologically more relevant—concentrations of CLE26p gave a reduction in primary root length and an increase in lateral root density, it was decided to focus on CLE26p for a more in-depth analysis of the sequence–activity relationship. To identify amino acids critical for CLE26p function, an alanine scan was performed using primary root length and lateral root density as biological assays (Fig. 4A–D; Supplementary Fig. S5 at *JXB* online). Seedlings grown on media containing 10 μM mCLE26p^R1A^, mCLE26p^P4A^, mCLE26p^R5A^, mCLE26p^G6A^, mCLE26p^D8A^, mCLE26p^P9A^, mCLE26p^I10A^, mCLE26p^H11A^, and mCLE26p^N12A^ showed no significant decrease in primary root length compared with untreated *A. thaliana*, and were significantly different from non-mutated CLE26p treatment (Fig. 4C). The total number of emerged lateral roots was similarly not decreased for these mCLE26p variants compared with the CLE26p control treatment (Supplementary Fig. S5). This suggested that these amino acid residues are critical for CLE26 function. In contrast, amino acid residues 2 and 3 do not appear to be critical for CLE function, since mCLE26p^K2A^ and mCLE26p^V3A^ displayed the same, but a less strong effect on primary root length as the non-mutated CLE26p variant (a decrease of between 52% and 63%) (Fig. 4C). Interestingly, mCLE26p^P7A^ displayed increased activity, with respect to both primary root length (a further decrease of 11%) and lateral root density (a further increase of 32%), compared with wild-type CLE26p (Fig. 4C, D). Finally, mCLE26p^D8A^ resulted in a slightly longer root than untreated seedling roots, but did not have an effect on lateral root density (Fig. 4C, D). Taken together, the series of alanine-substituted CLE26 peptides revealed several amino acids which are critical for bioactivity of CLE26, and pinpointed mCLE26p^P7A^ and mCLE26p^D8A^ as a hyperactive and a possible antagonistic peptide, respectively. Previous analyses of critical amino acid residues in, for example, CLE41/CLE44 [also referred to as TRACHEARY ELEMENT DIFFERENTIATION INHIBITORY FACTOR (TDIF)] (Ito *et al.*, 2006) and CLV3 (Kondo et al, 2008) identified similar amino acids that are important for the peptide to have the required bioactivity.

### Structural modelling of CLE26p explains the mCLE26p^P7A^ effect

**Fig. 5.**
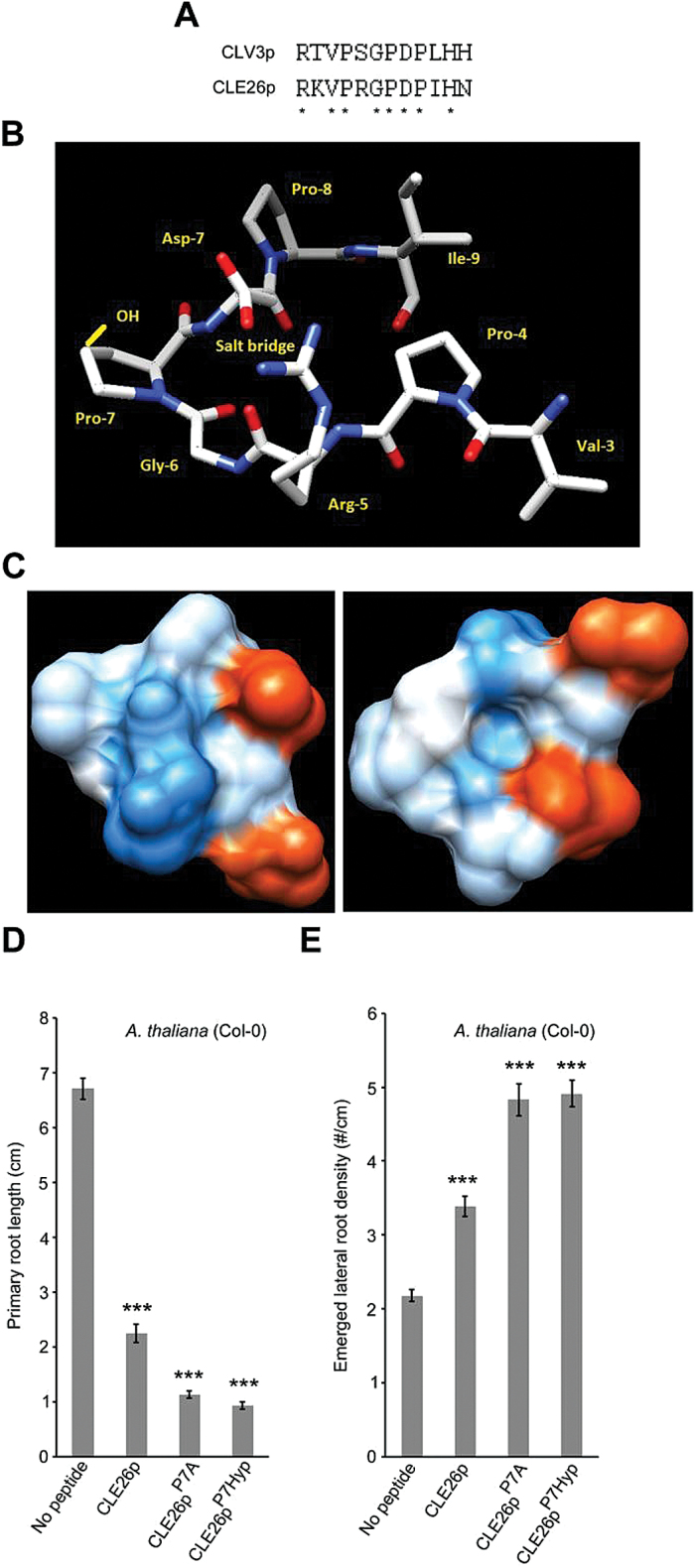
Sequence and structure versus activity of CLE26p. (A) Conserved residues between CLE26p and CLV3p are denoted by asterisks. (B) The top-ranked predicted structure with amino acids of the cleaved CLE26 peptide named, the position of a potentially stabilizing salt bridge marked, and the hydroxyl group of Pro-7-Hyp (in the 2S, 4S conformation reported from other studies) depicted in yellow. (C) The solvent-accessible surface (left) and solvent-accessible surface of the opposite face of the peptide in (B) (right) coloured in shades of red or blue to indicate the level of acidity or alkalinity, respectively. (D, E) Quantification of primary root length (D) and emerged lateral root density (E) for CLE26p, CLE27p^P7A^ (~mCLE26p A7), and CLE26p^7Hyp^-treated wild-type seedlings. The graph indicates the mean ±SE. Statistical significance (Student’s *t*-test) compared with no peptide treatment is indicated: ***P<0.01.

CLE26 has similar residues at the N- and C-termini as CLV3 that are critical for correct function in *A. thaliana*, and mutation of more central amino acids causes a similar reduction in bioactivity to that reported for CLV3 (Kondo *et al.*, 2008) (Fig. 5A). That these amino acids are critical for function indicates that they contribute to the correct conformation for ligand−receptor interaction. Structural analysis of CLE26 indicates that Gly6 and Pro7 form the sharp bend of a hairpin, potentially putting the proteolytic cleavage sites close together. This resembles earlier CLE structure predictions (Meng and Feldman, 2010). Rotamer analysis also showed that this conformation brings Arg5 and Asp8 into close proximity to form a salt bridge, potentially increasing the stability of the mature peptide (Fig. 5B). Surface hydrophobicity analysis suggested that one surface of this structure bulges and is predominantly basic, owing to Arg5, while the opposite surface is uncharged and has a cavity (Fig. 5C). These two surfaces could be involved in binding to receptors. The flanking precursor sequences are predicted to be α-helices, which may interact prior to mature peptide cleavage, facilitating the formation of a hairpin, and one of these could be membrane associated (Supplementary Fig. S7 at *JXB* online). It has been shown that CLV3p is hydroxylated on each of its proline residues and that the proline at position 7 in the CLV3 peptide is also arabinosylated, enhancing binding to CLV1 and CLV2 (Kondo *et al.*, 2006; Ohyama *et al.*, 2009; Shinohara and Matsubayashi, 2013). CLE26p also contains a proline residue at position 7, and it is worth noting that hydroxyl and arabinose side chains would point towards the arginine–aspartate surface noted above, perturbing their positions slightly (Fig. 5B). Similar modifications in CLE26p might be mimicked by mCLE26p^P7A^, potentially increasing binding affinity for its orphan receptor and explaining the enhanced biological activity. Indeed, the mCLE26p^P7A^ effect could be mimicked by using a CLE26p variant that is hydroxylated at position 7 (CLE26p^P7Hyp^) (Fig. 5D,E). The alanine at position 7 allows more flexibility in the arms of the hairpin, potentially enabling the arginine–aspartate surface to adopt a more favourable conformation for binding. Molecular dynamics studies of β-1,2-linked tri-arabinosylated CLV3p^P7^ suggest that the effect of the trisaccharide is to bring the N- and C-terminal ends of the peptide closer together (Shinohara and Matsubayashi, 2013), making it more like the hairpin model of CLE26.

The PXGPXP motif is conserved in most CLE peptides (Ito *et al.*, 2006), further indicating that formation of a bent hairpin structure is probably important for ligand–receptor interactions. Interestingly the highly conserved proline at position 7 is not critical for bioactivity of TDIF, CLV3, or CLE26. It is possible that the proline to alanine amino acid exchange at position 7 does not affect the peptide structure and thus does not affect activity, or that this conserved proline at position 7 is a target for post-translational modifications to ‘fine-tune’ the bioactivity of CLE peptides.

### AtCLE26p affects *Brachypodium* and wheat root architecture

**Fig. 6.**
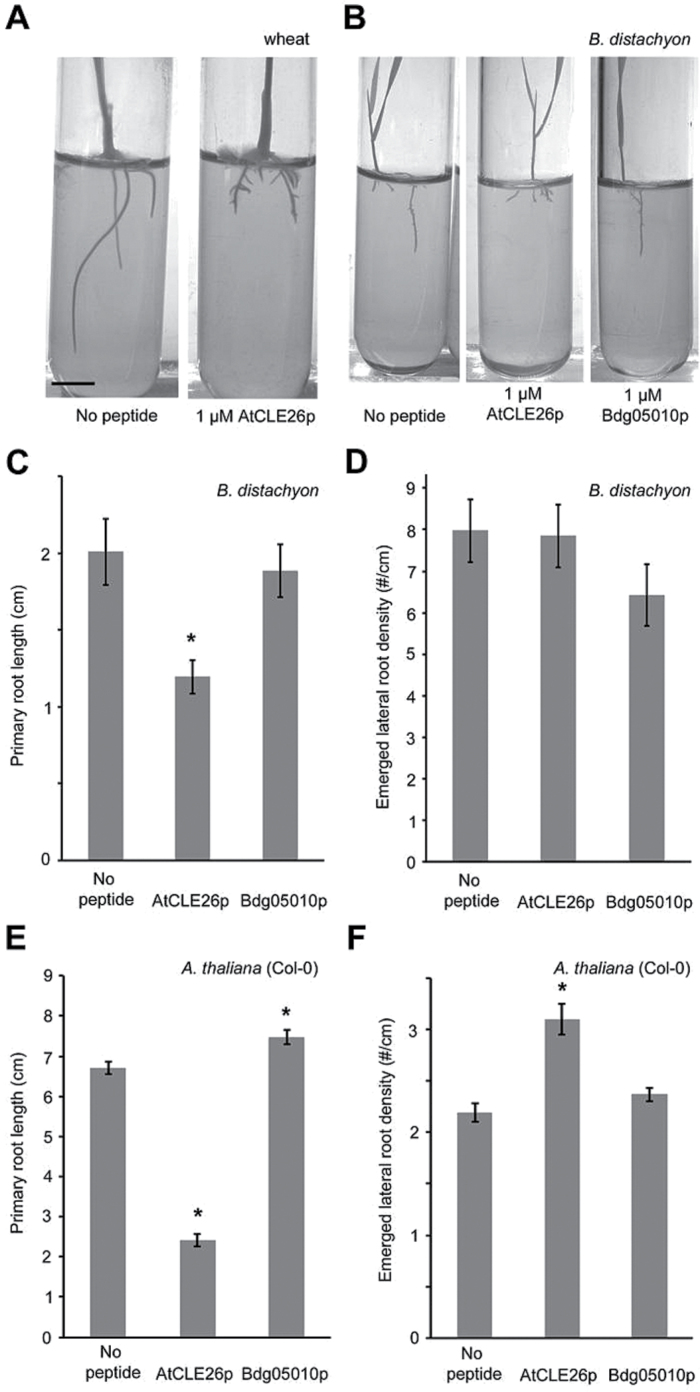
Effect of AtCLE26p and BdCLE26p on wheat, *B. distachyon,* and *A. thaliana.* (A, B) Representative pictures are shown for wheat (A) and *B. distachyon* (B) at 12 d after germination. (C, D) Quantification of *B. distachyon* seedling primary root length (C) and emerged lateral root density (D). (E, F) Quantification of *A. thaliana* seedling primary root length (C) and emerged lateral root density (D). The bar graphs indicate the mean ±SE. Statistical significance (Student’s *t*-test) compared with no peptide treatment is indicated: ***P<0.01. Scale bar=1cm.

To investigate whether the effect of CLE26p observed in *A. thaliana* also extends to monocots, the primary root length of CLE26p-treated *B. distachyon* (Bd21) and wheat was analysed. CLE26p treatment of *B. distachyon* and wheat resulted in a short primary root compared with the untreated control (Fig. 6A–C). These results were similar to those in *A. thaliana* and suggested that an orthologue of AtCLE26 may also be involved in regulating primary root growth in monocots. However, in contrast to *A. thaliana*, CLE26p application failed to induce any obvious change to lateral root density in *B. distachyon* (Fig. 6D).

**Fig. 7.**
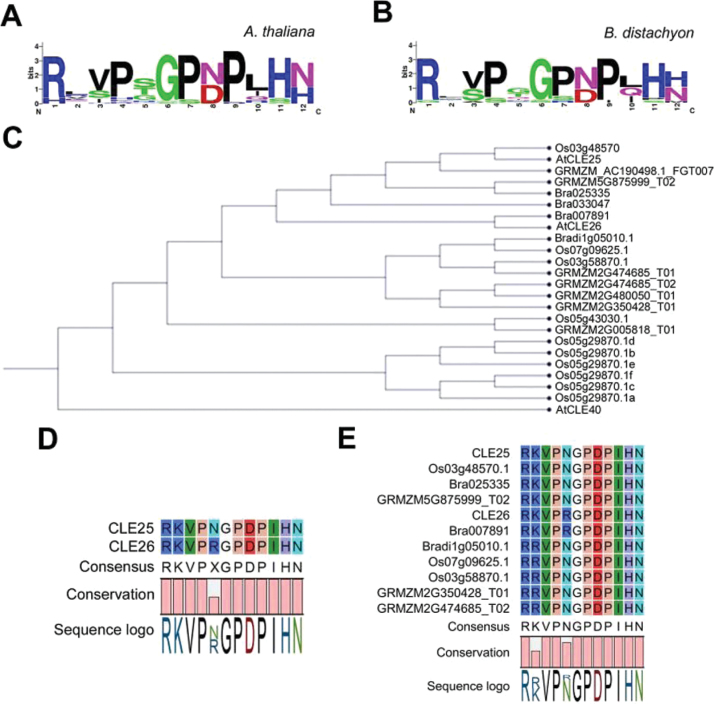
Phylogenetic analysis of mature CLE25 and CLE26 orthologues. (A, B) Weblogo for *A. thaliana* (A) and *B. distachyon* mature CLE peptide sequences (B). (C) Phylogenetic tree. (D-E) Alignment of the indicated CLE peptide sequences in *Arabidopsis thaliana*, *Oryza sativa* (Os), *Brassica rapa* (Bra), *Zea mays* (GRMZM), and *Brachypodium distachyon* (Bradi). (This figure is available in colour at *JXB* online.)

To explore CLE26 orthologues in monocots, CLE proteins were searched for in *Z. mays*, *O. sativa* and *B. distachyon*. A WebLogo alignment of mature peptide sequences from *A. thaliana* and *B. distachyon* indicated that mature CLE sequences are highly conserved, both within and between species (Fig. 7A, B). Phylogenetic analyses and sequence alignments indicated that *A. thaliana* mature CLE25 and CLE26 peptides are very similar and only differed at position 5 (asparagine and arginine, respectively) (Fig. 7C, D). While the CLE25p sequence appeared to be fully conserved in monocots such as rice and maize, completely conserved CLE26p sequences were not retrieved (Fig. 7C, E). Putative CLE26p-like sequences differed at one or two positions, namely position 2 (arginine instead of lysine) and 5 (asparagine instead of arginine) in *B. distachyon*, *Z. mays* and *O. sativa* (Fig. 7E). According to the blocks substitution matrix 62 (BLOSUM62), these substitutions are conservative amino acid substitutions—the substitution has similar biochemical properties to that of the original—and should, therefore, not strongly affect the bioactivity of the peptide. However, the CLE26p alanine scan showed that the arginine at position 5 was essential for function in *A. thaliana*, while the lysine at position 2 was not (Fig. 4). Based on these observations, it cannot be ruled out that a non-specific CLE26p effect was observed in wheat and *B. distachyon*, To investigate this further, a synthetic CLEp derived from a *B. distachyon Bradi1g05010*-encoded protein (Bd1g05010p), a CLE25p/CLE26p-related CLE peptide, was applied to *A. thaliana* seedlings. This did not recapitulate the effects observed for the *A. thaliana* CLE26p (Fig. 6E, F), indeed supporting that this peptide is not active in *A. thaliana*. In contrast, Bd1g05010p-treated *A. thaliana* seedlings display a slightly increased primary root length (Fig. 6E), suggesting that Bd1g05010p might act as an antagonistic peptide. Next, Bd1g05010p was applied to *B. distachyon* and the impact on root architecture was evaluated. Interestingly, Bd1g05010p did not reduce primary root length, but slightly decreased lateral root density (Fig. 6C, D). This is opposite to what was observed with the *A. thaliana* CLE26p and to what was previously observed for *A. thaliana* CLE25p (Strabala *et al.*, 2006; Kinoshita *et al.*, 2007). It appears that the change of the conserved amino acid K into R has a strong impact on functionality. While this needs to be explored in more detail, the monocot Bd1g05010p is possibly not a functional orthologue of *A. thaliana* CLE26p or even CLE25p. This may indicate the presence of another (currently unknown) peptide present in *B. distachyon*, which, although less similar in primary sequence to *A. thaliana* CLE26p, is able to form a similar secondary structure, fulfilling the role of CLE26 role in *B. distachyon.*

### CLE26 peptide affects auxin response

**Fig. 8.**
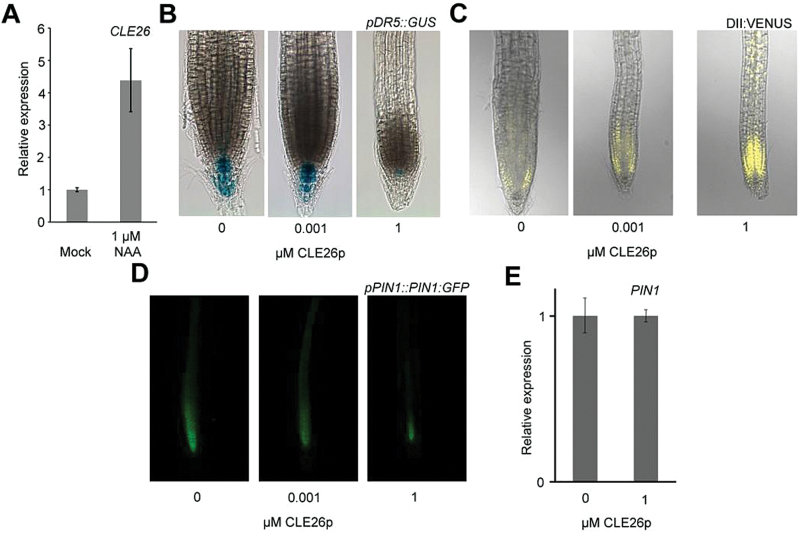
CLE26 and auxin response/transport. (A) *CLE26* expression level as determined by qPCR in auxin-treated (6h) 5-day-old wild-type seedlings. (B–D) CLE26p-treated *pDR5::GUS* (B), *35S::DII:VENUS* (C), and *pPIN1::PIN1:GFP* 5-day-old seedlings continuously grown on CLE26p (D). (E) *PIN1* expression level in 7-day-old seedling roots treated with CLE26p for 24h. The bar graph indicates the mean ±SE.

Since auxin plays a dominant role in primary root growth and lateral root initiation and development (De Smet, 2012; Lavenus *et al.*, 2013; Tian *et al.*, 2014), it was explored whether and potentially where CLE26p would have an influence on auxin response. For this, it was first tested if *CLE26* expression was regulated by auxin. qPCR analyses showed an ~4-fold increase in *CLE26* expression in wild-type seedling roots following 6h of auxin treatment (Fig. 8A). Subsequently, it was tested whether CLE26p affects the auxin response marker *pDR5::GUS*. At 1nM CLE26p, a concentration that significantly affected primary root growth, no obvious difference in *pDR5::GUS* expression was observed in the primary root tip (Fig. 8B). However, at a higher concentration (1 μM), the *pDR5::GUS* expression level was significantly reduced (Fig. 8B). Subsequently, the AUX/IAA protein-based auxin sensor *p35S::DII:VENUS* (Vernoux *et al.*, 2011; Brunoud *et al.*, 2012) was tested upon CLE26p treatment. While a mild increase in DII:VENUS fluorescence was observed at 1nM CLE26p, there was a dramatic increase at 1 μM (Fig. 8C). In agreement with the *pDR5::GUS* results (Fig. 8B), this suggested an altered auxin response in the root tip. These observations are similar to what was recently observed using 10nM CLE26p on *pDR5::NLS-3xVENUS* for 48h (Rodriguez-Villalon *et al.*, 2015). Next, to determine if the effect of CLE26p on the auxin response and/or distribution in the root tip could be related to CLE26p regulation of polar auxin transport, the *pPIN1::PIN1:GFP* marker line was used (Benková *et al.*, 2003). The *pPIN1::PIN1:GFP* marker displayed mildly and strongly reduced fluorescence at 1nM and 1 μM, respectively (Fig. 8D; Supplementary Fig. S8 at *JXB* online). An even stronger effect on PIN1 fluorescence and localization was observed with 1nM CLE26p^P7Hyp^ (Supplementary Fig. S8). However, this reduction is in contrast to the qPCR results, where CLE26p treatment did not dramatically affect *PIN1* expression in the root (Fig. 8E). This suggested a possible CLE26p-mediated effect on PIN1:GFP at the protein level, which could explain the reduced auxin response in the root tip.

**Fig. 9.**
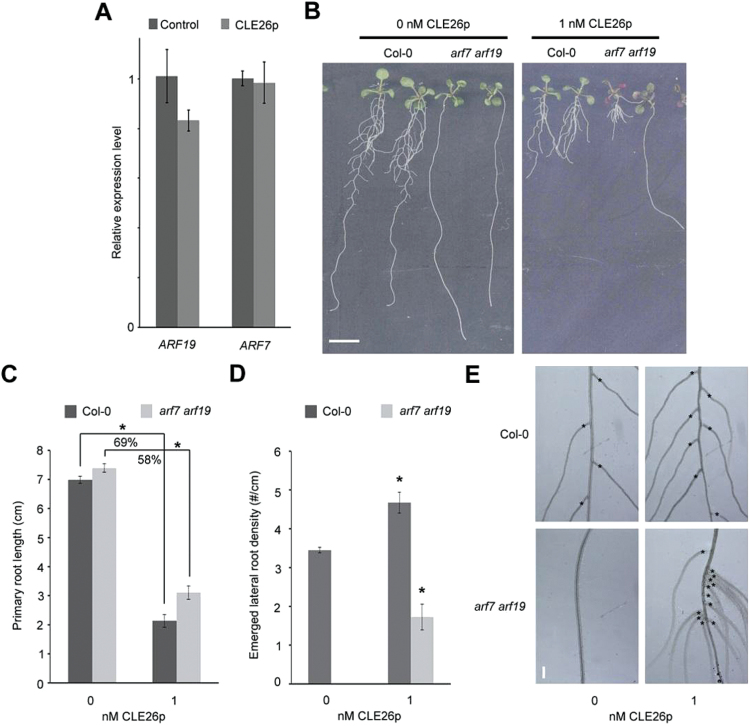
CLE26p and ARF7−ARF19. (A) *ARF7* and *ARF19* expression as determined by qPCR in 7-day-old seedling roots treated with 1 μM CLE26p for 24h. The bar graph indicates the mean ±SE. (B–E) Root phenotype of CLE26p-treated Col-0 and *arf7arf19* at 9 d after germination. Representative pictures (B) and quantification of primary root length (C) and emerged lateral root density (D). The bar graphs indicate the mean ±SE. Statistical significance (Student’s *t*-test) compared with no peptide treatment: *P<0.05. Scale bar=1cm (B) and 100 μm (E). (E) Detail of lateral root positions (asterisk) and density in Col-0 and *arf7arf19* following peptide treatment. (This figure is available in colour at *JXB* online.)

Finally, it was analysed whether CLE26p acts upstream or downstream of the ARF7–ARF19 module, a major regulator of lateral root development (Lavenus *et al.*, 2013). For this, the impact of CLE26p treatment on *ARF7* and *ARF19* expression was tested using qPCR. In CLE26p-treated roots, the *ARF7* and *ARF19* expression levels are not significantly affected (Fig. 9A). Subsequently, the effect of CLE26p treatment on *arf7arf19* was tested. Interestingly, the lateral rootless *arf7arf19* double mutant was partially insensitive to CLE26p with respect to primary root length, but displayed a limited—often closely grouped—number of lateral roots upon CLE26p treatment (Fig. 9B–E). In conclusion, on the one hand, CLE26p appears to require an ARF7- and ARF19-dependent auxin response for its activity, but, on the other hand, it appears to be able to induce lateral root development in *arf7arf19*. These two—seemingly opposing—effects can possibly be reconciled through a CLE26-mediated perturbation of auxin transport and accumulation.

### An *Arabidopsis cle26* mutant affects root architecture

**Fig. 10.**
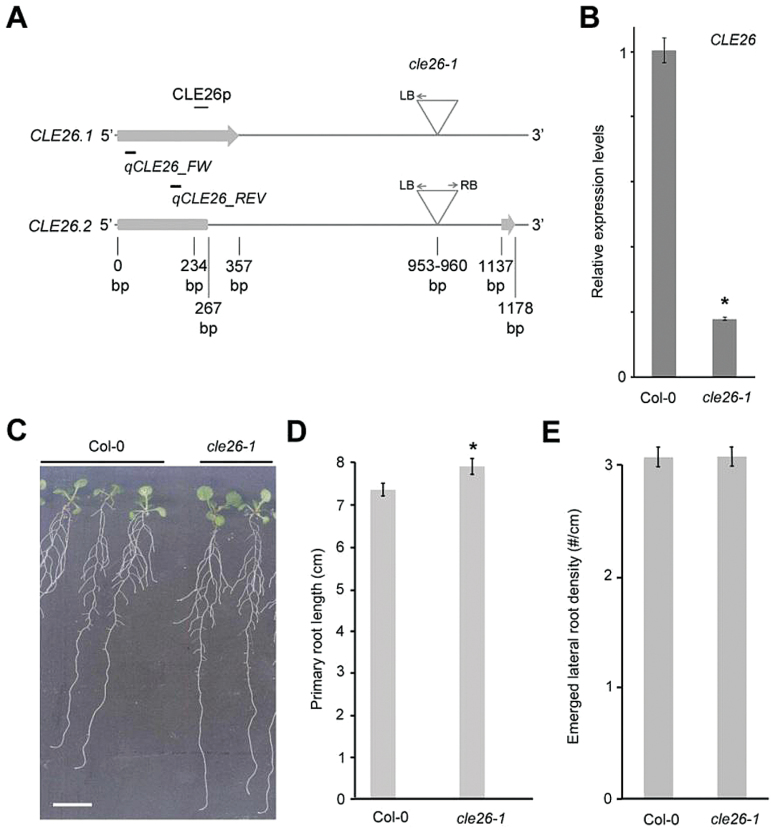
Characterization of the *cle26-1* mutant. (A) Position of the T-DNA insertion in two splicing variants. Primers used in qPCR are indicated. (B) *CLE26* expression level as determined by qPCR in *cle26-1* roots. (C–E) Root phenotype of *cle26-1* at 9 d after germination. Representative pictures of wild-type and *cle26-1* seedlings (C). (D, E) Quantification of primary root length (D) and emerged lateral root density (E) for *cle26-1* seedlings. The graphs indicate the mean ±SE. Statistical significance (Student’s *t*-test) compared with Col-0 is indicated: *P<0.05. Scale bar=1cm. (This figure is available in colour at *JXB* online.)

To explore further the role of CLE26 in mediating root architecture, a T-DNA insertion line, which was named *cle26-1*, was analysed (Fig. 10A). Interestingly, *CLE26* has two splicing variants that are both expressed in the root (Fig. 10A; Supplementary Fig. S9 at *JXB* online). qPCR was performed to examine *CLE26* mRNA transcript levels (using a primer pair that captures both *CLE26.1* and *CLE26.2*) in *cle26-1*, and an ~80% decrease in *CLE26* expression was observed (Fig. 10B). Then the primary root length and lateral root density of *cle26-1* were analysed. This revealed a mild, significant increase with respect to primary root length, and no difference in the level of emerged lateral root density (Fig. 10C–E). Overall, it appears that CLE26 impacts on primary root length and that its effect on lateral root density may be compensated for by other closely related signalling pathways or by genetic redundancy with other CLE peptides.

### Conclusion

Taken together, the gain- and loss-of-function data support a role for CLE26 in regulating root architecture. It was recently reported that both CLE45 and CLE26 affect primary root protophloem differentiation, which in turn systemically affects root branching (Rodriguez-Villalon *et al.*, 2014, 2015). The present results on CLE26 confirm, and are complementary to, these recently published data on the *CLE26* expression pattern and the CLE26p effect on auxin response and *Arabidopsis* root system architecture. The findings indicate that application of CLE26p decreases the distribution of auxin to the root apical meristem, seemingly by decreasing the abundance of PIN1 through post-translational regulation. Interestingly, it has been reported that localization of PIN1 is responsive to cytokinin signalling (Bishopp *et al.*, 2011). Since CLE14 and CLE20 have previously been demonstrated to be cytokinin responsive (Meng and Feldman, 2010), it will be of interest to determine whether CLE26 interacts with cytokinin signalling, which may indicate a role for CLE26 in mediating localization and/or degradation of PIN1 in response to cytokinin (Marhavy *et al.*, 2014). However, it is also possible that altered CLE26-mediated protophloem differentiation globally affects shoot to root transport and/or protein localization, including PIN1-mediated auxin distribution, but also, for example, the distribution of sugars.

On a structural level, the alanine scanning data indicated a hyperactive proline (CLE26^P7^). Further analysis indicated that the mCLE26p^P7A^ variant mimics the effect on bioactivity of CLE26p^P7Hyp^. The former could affect the position of the arms of the loop, while the latter could achieve the same effect by altering the positions of Arg5 and Asp8. The CLE26 model also suggests that other interactions between amino acids may contribute to the folding process, and thus the bioactivity of CLE26p. In view of a possible antagonistic peptide, a new approach to obtain loss-of-function phenotypes that has, however, some limitations (Song *et al.*, 2013; Czyzewicz *et al.*, 2015), mCLE26p^D8A^ has some potential as it appears to mimic the *cle26-1* phenotype.

In monocot model systems (*Brachypodium* and wheat), synthetic CLE26 application exhibited a similar reduction in primary root length, suggesting an orthologous signalling pathway. However, application of a sequentially orthologous peptide (termed Bd1g05010p) to *Brachypodium* did not induce a short-root phenotype. This suggests that *Brachypodium* may have evolved a separate signalling peptide to fulfil the same role as CLE26, that, although less similar in primary sequence, presumably forms a similar functional secondary structure. Similarly, synthetic Bd1g05010p did not induce a short-root phenotype when applied to *Arabidopsis* at the same concentration as AtCLE26p, indicating that the peptide is either non-functional, or requires a higher concentration in order to stimulate its receptor in both species.

## Supplementary data

Supplementary data are available at *JXB* online.

Figure S1. *pCLE::GUS* expression in *Arabidopsis* primary root tip.

Figure S2. *pCLE::GUS* expression during early stages of *Arabidopsis* lateral root development.

Figure S3. *pCLE::GUS* expression during later stages of *Arabidopsis* lateral root development.

Figure S4. *pCLE::GUS* expression in selected stages of lateral root development.

Figure S5. Emerged lateral root numbers corresponding to Figs 2D, E, and 4D.

Figure S6. CLE26p effect on *pAT2G18380::GFP* in primary root tip.

Figure S7. CLE26 consensus secondary structure prediction.

Figure S8. CLE26p effect on PIN1:GFP.

Figure S9. *CLE26* splicing variants in the root.

Figure S10. PSI-BLAST output for CLE26.

Table S1. *pCLE::GUS* lines.

Supplementary Data
