# Supplementary material for: Modulation of Arabidopsis and monocot root architecture by CLAVATA3/EMBRYO SURROUNDING REGION 26 peptide
Source: J Exp Bot. 2015 Jul 17;66(17):5229–43. doi: 10.1093/jxb/erv360 (PMC4526925; doi:10.1093/jxb/erv360)
Supplement: Supplementary Data [file supp_66_17_5229__index.html]

Modulation of Arabidopsis and monocot root architecture by CLAVATA3/EMBRYO SURROUNDING REGION 26 peptide — Supplementary Data 

# Modulation of *Arabidopsis* and monocot root architecture by CLAVATA3/EMBRYO SURROUNDING REGION 26 peptide

## Supplementary Data

Data files

- Supplementary Data - Supplementary Data
