# Supplementary material for: Modulation of Arabidopsis and monocot root architecture by CLAVATA3/EMBRYO SURROUNDING REGION 26 peptide
Source: J Exp Bot. 2015 Jul 17;66(17):5229–43. doi: 10.1093/jxb/erv360 (PMC4526925; doi:10.1093/jxb/erv360)
Supplement: Supplementary Data [file supp_erv360_jexbot147967_file001.pdf]

Modulation of Arabidopsis and monocot root architecture by CLAVATA3/EMBRYO SURROUNDING REGION 26 peptide

Nathan Czyzewicz, Chun-Lin Shi, Lam Dai Vu, Brigitte Van De Cotte, Charlie Hodgman, Melinka A. Butenko, and Ive De Smet

SUPPLEMENTARY INFORMATION

Supplemental methods

Structure prediction

The first phase carried out by PHYRE involves determining the consensus of several secondary-structure prediction algorithms (**Supplementary Fig. S7**). The helical N-terminal signal peptide is clearly predicted. The putative region of transmembrane helix is too small to cross a membrane, but could enable the precursor protein to be membrane-associated. In the next phase of structure prediction, the input sequence was used to query the protein sequence databases using PSI-BLAST to build up as large a multiple sequence alignment as possible. In this instance, three cycles of BLASTP searches were necessary. The output of this query is shown in **Supplementary Fig. S10**. The alignment is used to generate a Hidden Markov Model that is used to query all the structures in the Protein DataBank. The structures are ranked and threaded structure models of the CLE26 sequence produced. The ranked structures are as follows (N.B. the mature peptide covers positions 78-89):

Template	CLE26 sub-sequence position	Confidence	Percentage identity to template
c3u02C	80 - 87	36.1	50
c3bcvA	86 – 115	20.9	27
c3czdA	52-86	20.4	31
c3fynH	70 - 106	19.3	32

All structures predicted for the mature peptide corresponded to a loop as expected from the number of proline residues, with the model derived from c3fynH having interacting flanking

helices. Only the top-ranked model was taken for further study because it contained the core region of the peptide, unlike the 2nd and 3rd ranked models, and there was very little confidence in the 4th ranked model. The complete prediction dataset is available from the authors upon request. Plots of the N and C-terminal flanking helices show that the former has a hydrophobic surface suitable for helix-helix packing and the latter is so hydrophobic that it could prefer to bind to the surface of a membrane.

Supplemental Tables and Figures

Supplementary Table S1 – *pCLE::GUS* lines

Line	Type	Source
pCLE1::GUS	GUS Reporter	NASC – N66264
pCLE2::GUS	GUS Reporter	NASC – N66266
pCLE3::GUS	GUS Reporter	NASC – N66268
pCLE4::GUS	GUS Reporter	NASC – N66270
pCLE5::GUS	GUS Reporter	NASC – N66272
pCLE6::GUS	GUS Reporter	NASC – N66274
pCLE7::GUS	GUS Reporter	NASC – N66276
pCLE8::GUS	GUS Reporter	NASC – N66278
pCLE9::GUS	GUS Reporter	NASC – N66280
pCLE10::GUS	GUS Reporter	NASC – N66283
pCLE11::GUS	GUS Reporter	NASC – N66286
pCLE12::GUS	GUS Reporter	NASC – N66288
pCLE13::GUS	GUS Reporter	NASC – N66290
pCLE16::GUS	GUS Reporter	NASC – N66294
pCLE17::GUS	GUS Reporter	NASC – N66296
pCLE18::GUS	GUS Reporter	NASC – N66298
pCLE21::GUS	GUS Reporter	NASC – N66302
pCLE22::GUS	GUS Reporter	NASC – N66304
pCLE25::GUS	GUS Reporter	NASC – N66306
pCLE26::GUS	GUS Reporter	NASC – N66308
pCLE27::GUS	GUS Reporter	NASC – N66310

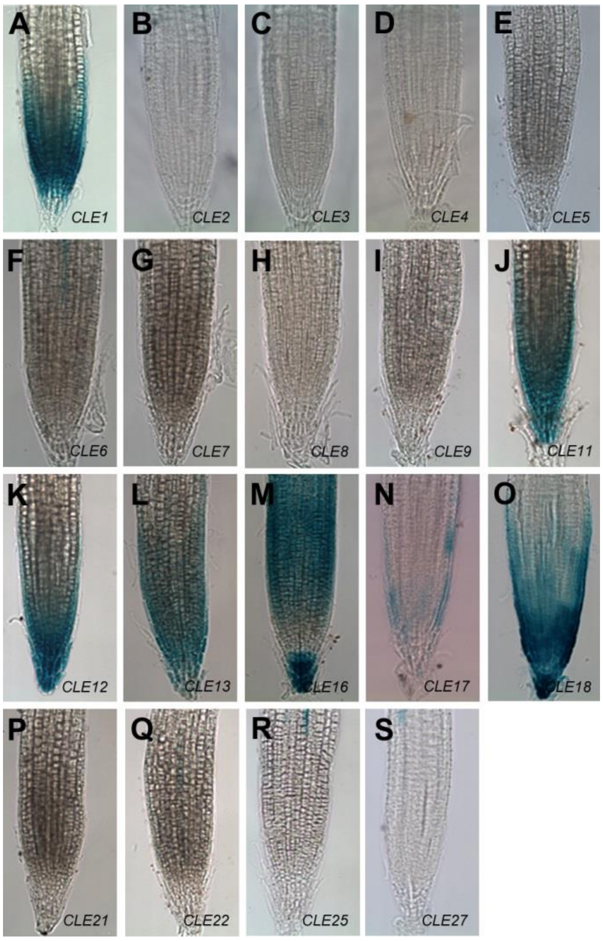

Supplementary Figure S1. *CLE* expression in *Arabidopsis* primary root tip visualised through *pCLE::GUS* lines.

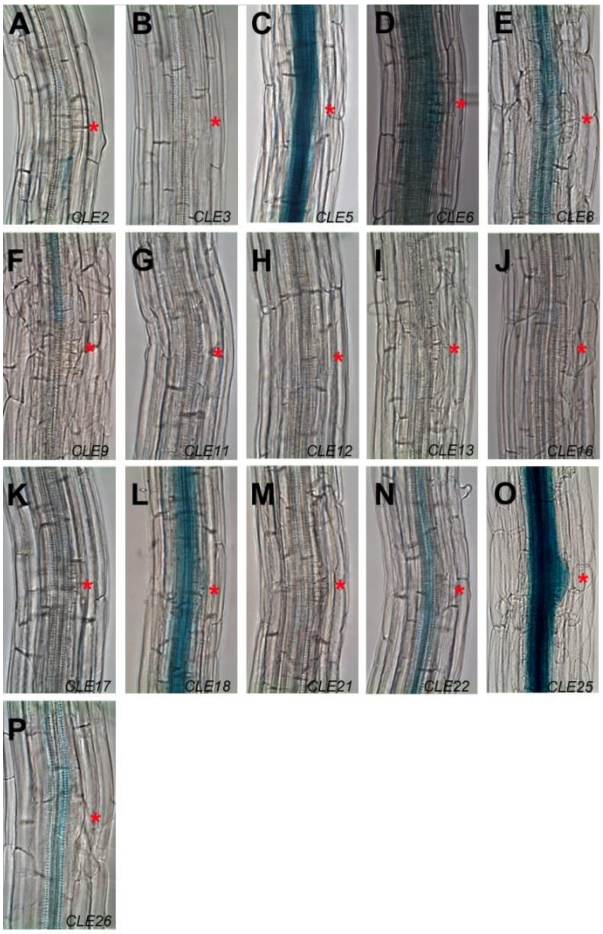

Supplementary Figure S2. *CLE* expression during early stages of *Arabidopsis* lateral root development visualised through *pCLE::GUS* lines. Red asterisk, position of lateral root primordium.

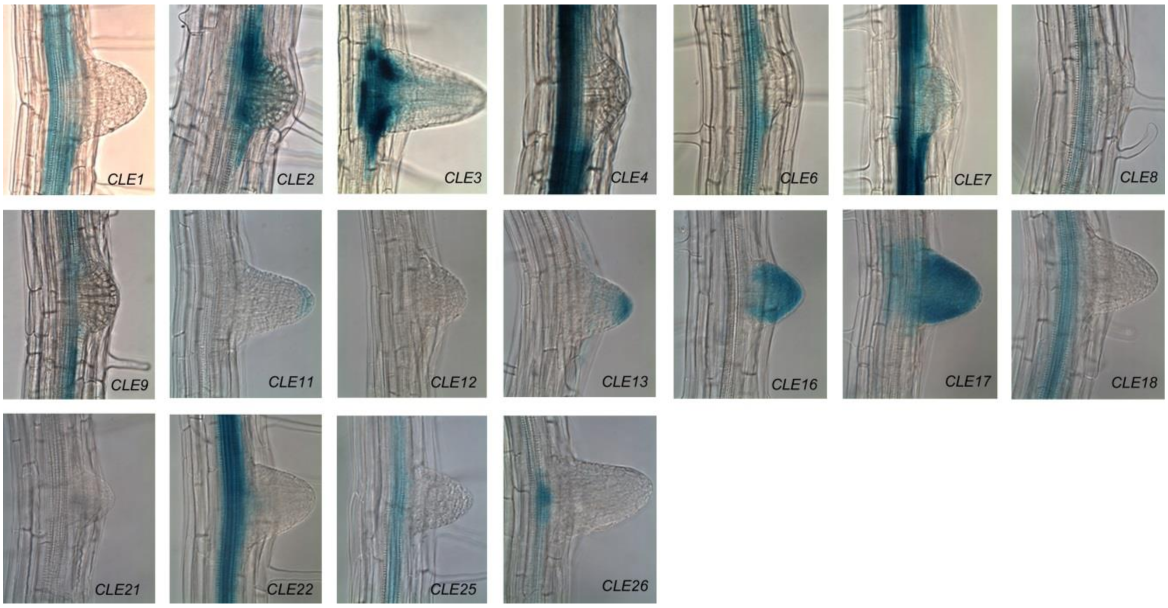

Supplementary Figure S3. *CLE* expression during later stages of *Arabidopsis* lateral root development visualised through *pCLE::GUS* lines. Red asterisk, position of lateral root primordium.

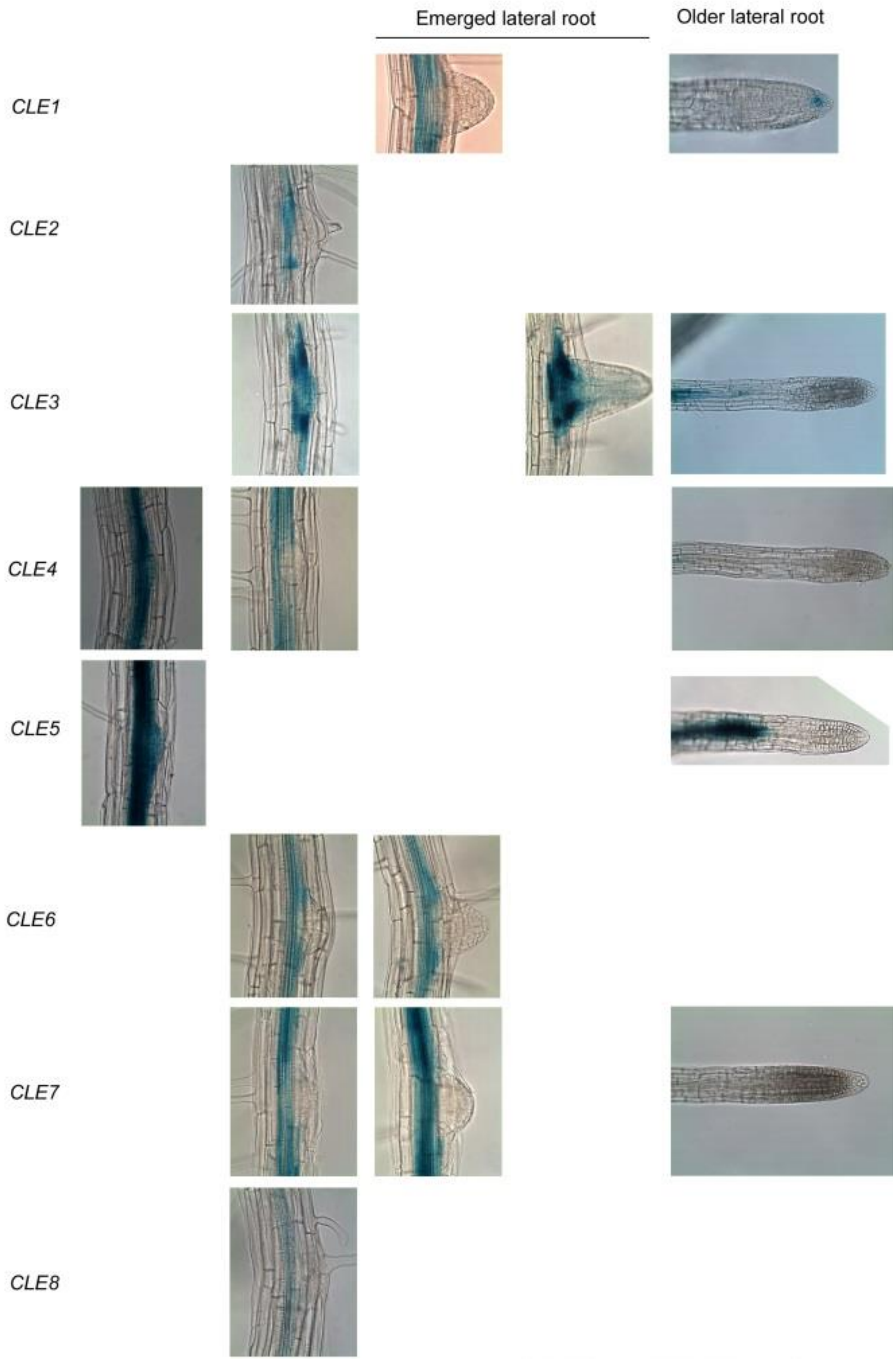

Supplementary Figure S4. *pCLE::GUS* expression in selected stages of lateral root development. Representative images are shown.

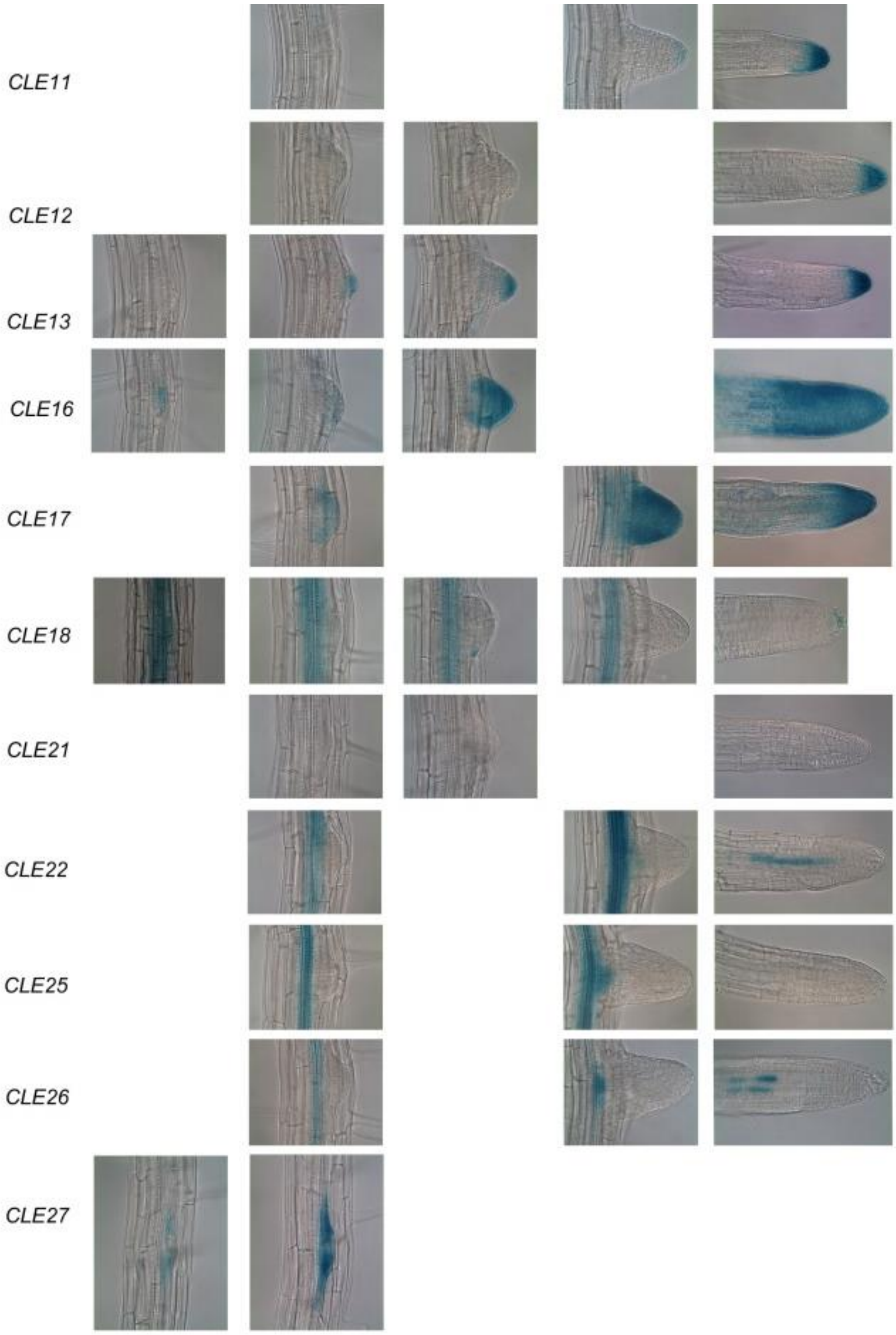

Supplementary Figure S4 (continued). *pCLE::GUS* expression in selected stages of lateral root development. Representative images are shown.

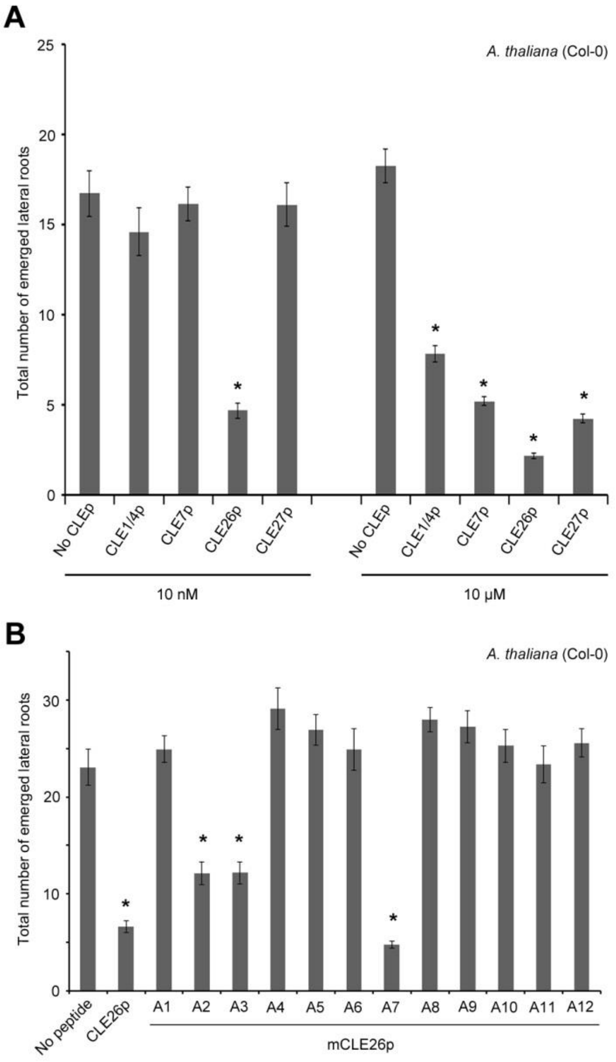

Supplementary Figure S5. Emerged lateral root numbers corresponding to Figure 2D and E (A) and Figure 4D (B). The bar graphs indicate the mean ± standard error. Statistical significance (Student's *t*-test) compared to the no peptide treatment is indicated: *, p-value < 0.01.

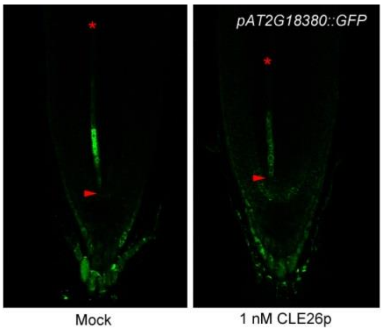

Supplementary Figure S6. *pAT2G18380::GFP* in primary root tip of seedlings 5 days after germination grown on ½ MS containing mock (left) or 1 nM CLE26p (right). Red asterisk and arrowhead point to position of (proto)phloem.

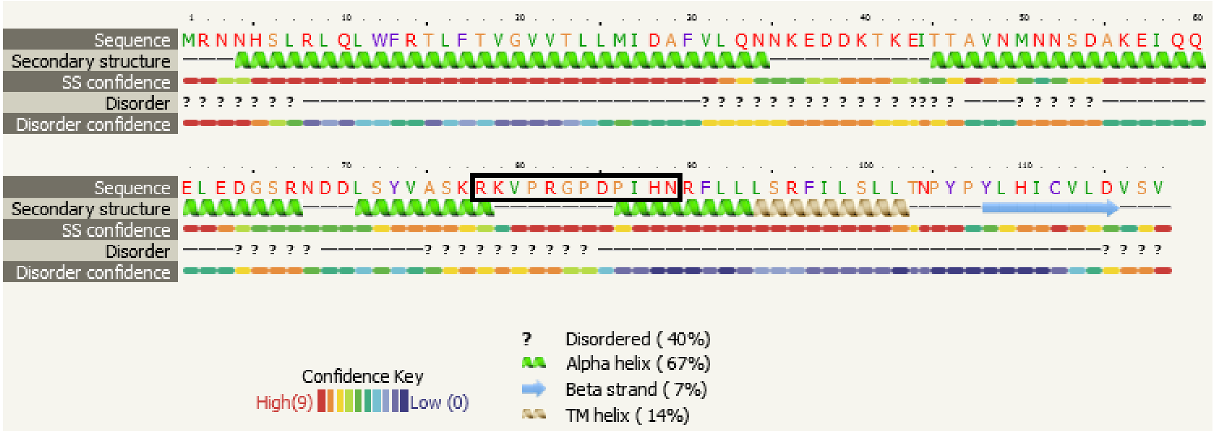

Supplementary Figure S7. Consensus secondary structure prediction. The CLE26 amino-acid sequence is shown, in which each residue is colour coded by the biophysical properties of its side chain. Below the sequence is the consensus secondary structure prediction with confidence codes and a prediction of disordered regions with confidence codes. The mature CLE26 peptide is denoted by the black box.

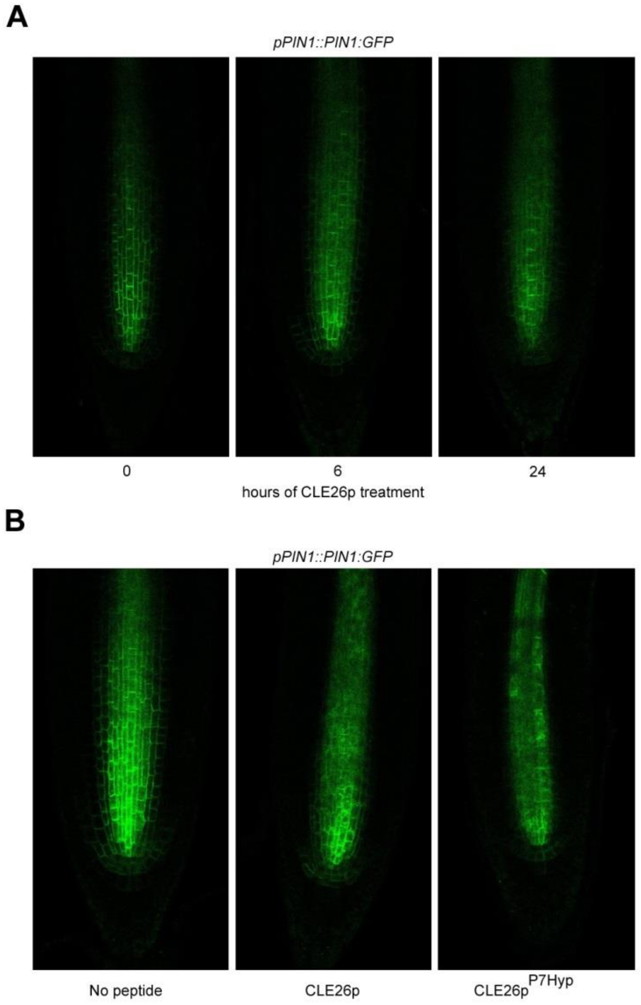

Supplementary Figure S8. Effect on PIN1:GFP by CLE26p treatment. (A) Different treatment length (hours) using 1 µM CLE26p on 5-day-old seedlings expressing *pPIN1::PIN1:GFP*. **(B)** 5-day-old seedlings expressing *pPIN1::PIN1:GFP* continuously grown on 1 nM CLE26p or CLE26p^{P7Hyp}.

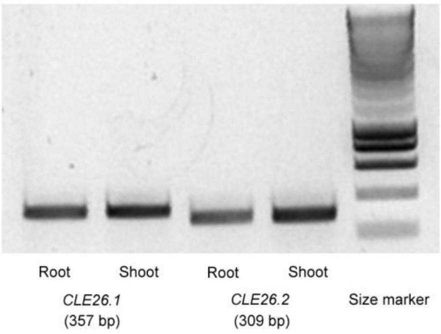

Supplementary Figure S9. *CLE26* splicing variants in the root. Both splicing variants can be detected in wild type (Col-0) roots.

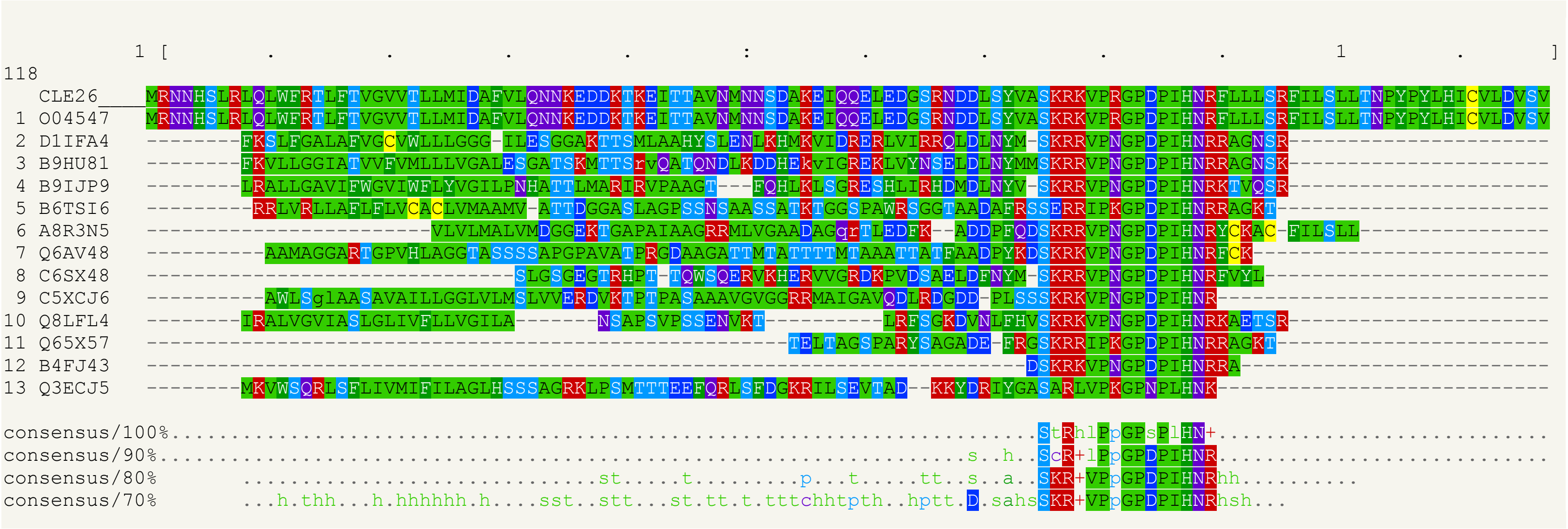

Supplementary Figure S10. PSI-BLAST Output. Below the query CLE26 sequence, the retrieved sequences are shown in order of similarity and BLASTP cycle. The consensus sequences at 4 different stringencies are also shown. Amino acids have been coloured by their biophysical properties. Image generated using MView 1.51.1.
